# Supplementary material for: Electrosynthesis of a nylon-6 precursor from cyclohexanone and nitrite under ambient conditions
Source: Nat Commun. 2023 May 26;14:3057. doi: 10.1038/s41467-023-38888-6 (PMC10219941; doi:10.1038/s41467-023-38888-6)
Supplement: Supplementary file 1 — Supplementary Information [file 41467_2023_38888_MOESM1_ESM.pdf]

Supplementary Information

Electrosynthesis of a nylon-6 precursor from cyclohexanone and nitrite under ambient conditions

Wu et al.

Contents

Supplementary Figure 1. The conventional routes for cyclohexanone oxime production.

Supplementary Figure 2. SEM image of Cu-S.

Supplementary Figure 3. XPS image of Cu-S.

Supplementary Figure 4. Reaction setup.

Supplementary Figure 5. Linear sweep voltammetry (LSV) studies of Cu-S in different electrolytes.

Supplementary Figure 6. ^{1}H NMR spectra of cyclohexanone oxime.

Supplementary Figure 7. ^{13}C NMR spectra of cyclohexanone oxime.

Supplementary Figure 8. GC–MS spectra of cyclohexanone oxime.

Supplementary Figure 9. Standard calibration curves for quantitative analysis of cyclohexanone cyclohexanone oxime.

Supplementary Figure 10. Potential-dependent cyclohexanone oxime FE over a Cu-S cathode.

Supplementary Figure 11. Standard calibration curves for quantitative analysis of NH_4^+.

Supplementary Figure 12. Potential-dependent NH_4^+ yield and FE over a Cu-S cathode.

Supplementary Figure 13. Potential-dependent cyclohexanone conversion, cyclohexanone oxime yield, selectivity, and FE over a Cu cathode.

Supplementary Figure 14. Potential-dependent NH_4^+ yield and FE over a Cu cathode.

Supplementary Figure 15. Cyclohexanone oxime yield and FE under the conditions of different molar ratios of cyclohexanone and NO_2^-.

Supplementary Figure 16. SEM image of Cu-S catalyst after stability test.

Supplementary Figure 17. XPS of Cu-S catalyst after stability test.

Supplementary Figure 18. Free energy diagram for cyclohexanone oxime generation over a Cu-S cathode.

Supplementary Figure 19. The optimized adsorption geometries of NO_2^- reduction intermediate on the Cu-S surface.

Supplementary Figure 20. FEs and yields of NO_2^- for NH_4^+ electrooxidation over a $Cu(OH)_2$ electrode.

Supplementary Figures 21~33. ^{1}H and ^{13}C NMR spectra of oximes products.

Supplementary Figure 34. Photograph of flow reactor setup and schematic illustration of electrolyzer.

Supplementary Figure 35. Performance of electrochemical synthesizing cyclohexanone oxime using a flow electrolyzer.

Supplementary Note 1. Analysis of the XPS results of Cu-S .

Supplementary Notes 2~3. NMR spectra of cyclohexanone oxime.

Supplementary Note 4. Reaction conditions for cyclohexanone oxime electrosynthesis over a Cu-S cathode.

Supplementary Note 5. Reaction conditions for cyclohexanone oxime electrosynthesis over a Cu cathode.

Supplementary Note 6. Techno-economic analysis (TEA) details.

Supplementary Note 7. Calculation details.

Supplementary Note 8. Experiment detailes for the electrooxidation of NH_4^+ to NO_2^-

Supplementary Note 9~21. NMR spectra of cyclohexanone oximes products.

Supplementary Note 22. Flow electrolyzer details.

Supplementary References (1-13)

Supplementary Figures

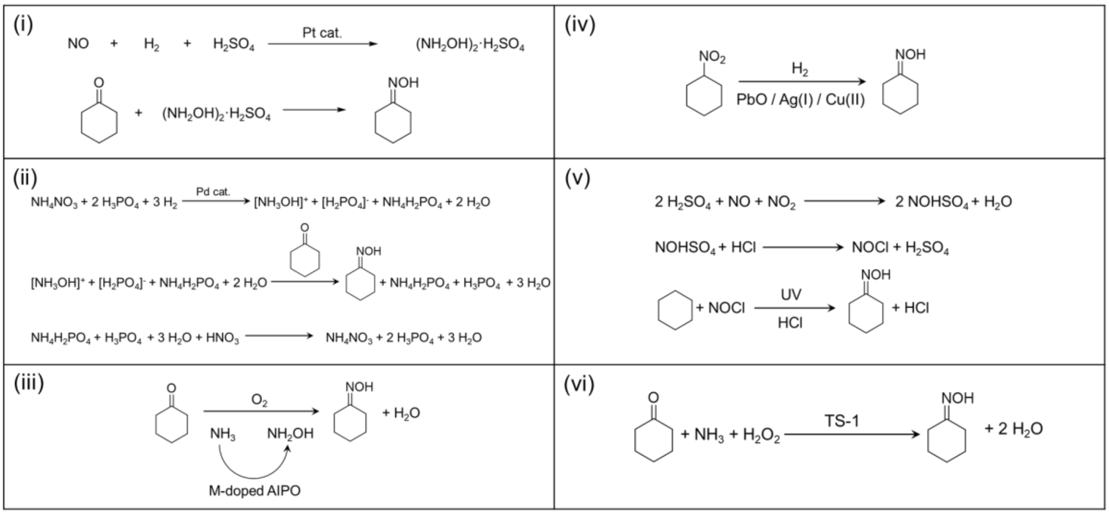

Supplementary Figure 1. (i, ii) The conventional route, which combines cyclohexanone with hydroxylamine, generated from either (i) hydroxylamine sulfate or (ii) hydroxylamine phosphate. (iii) The in situ synthesis of hydroxylamine from O_2 and ammonia over metal (M)-doped AlPO. (iv) The hydrogenation of nitrocyclohexane over homogenous salts. (v) The photonitrosation of cyclohexane. (vi) The ammoximation route, which produces hydroxylamine in situ through the combination of H_2O_2 and NH_3 over titanosilicate TS-1[1].

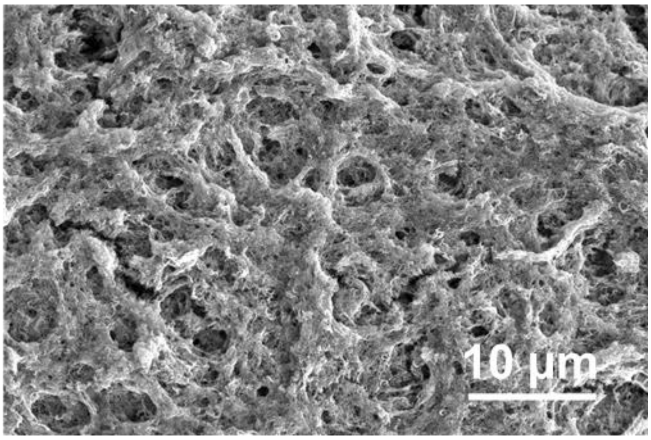

Supplementary Figure 2. SEM image of Cu-S catalyst before reaction.

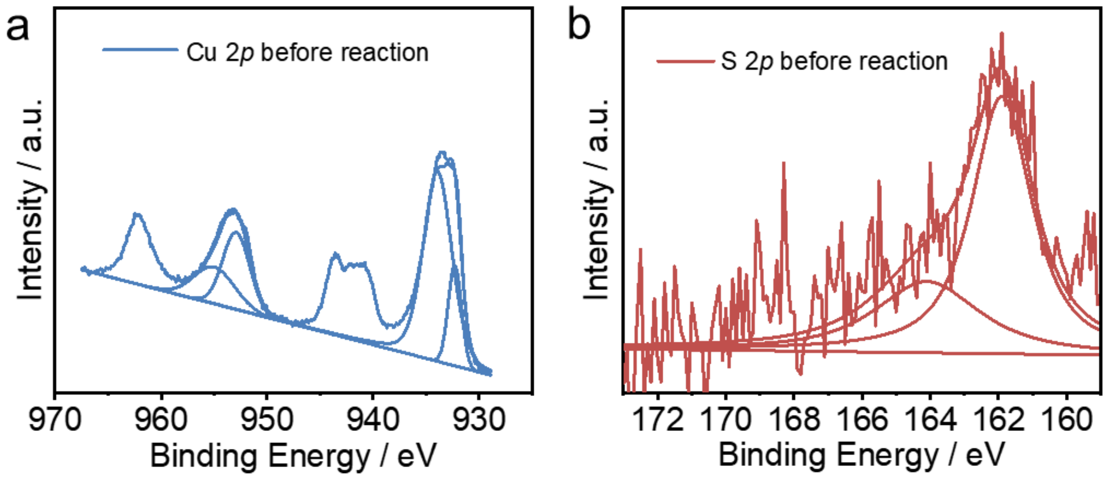

Supplementary Figure 3. Cu 2*p* and S 2*p* spectra of the Cu-S catalyst before the reaction.

Supplementary Note 1. The peaks at 932.2 and 953 eV were assigned to the Cu^{+} $2p_{3/2}$ and Cu^{+} $2p_{1/2}$ of Cu_2O, whereas the small peaks at 933.5 eV and 954.1 with the satellite peak at 942 eV correspond to the Cu^{2+} of CuO because of its oxidation in air.

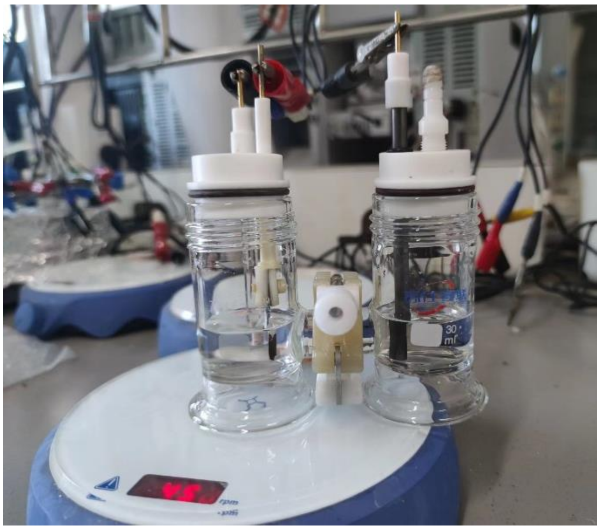

Supplementary Figure 4. Reaction setup for the electrochemical synthesis of cyclohexanone oxime over a Cu-S cathode.

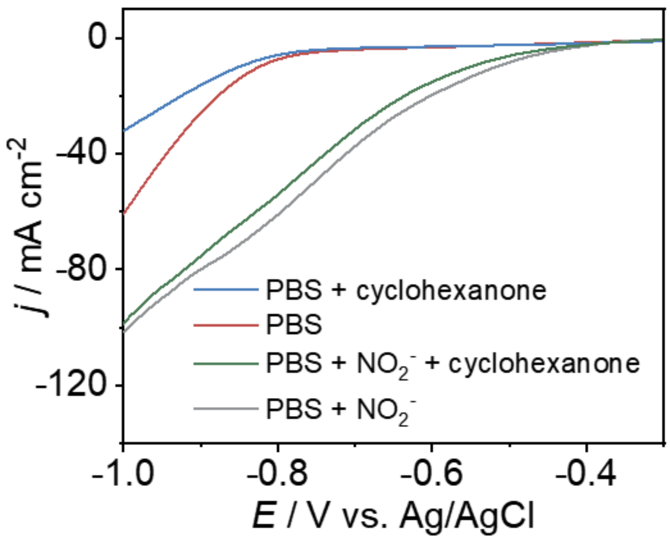

Supplementary Figure 5. LSV curves of Cu-S in different electrolytes.

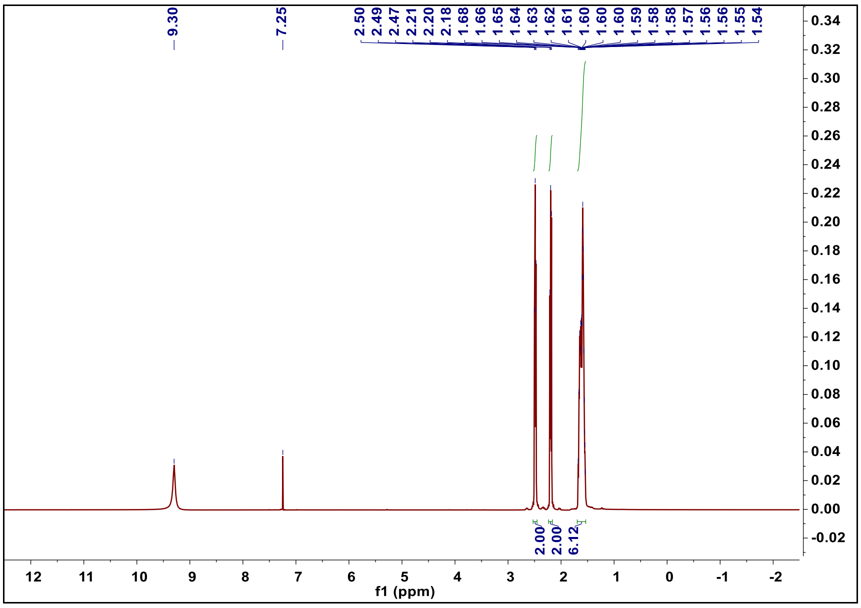

Supplementary Figure 6. ^{1}H NMR spectra of the cyclohexanone oxime product.

Supplementary Note 2. ^{1}H NMR (400 MHz, Chloroform-*d*) δ [ppm] 8.99 (s, 0.95H), 2.50 (t, *J* = 6.0 Hz, 2H), 2.21 (t, *J* = 6.0 Hz, 2H), 1.68-1.56 (m, 6H). GC/MS 113.0.

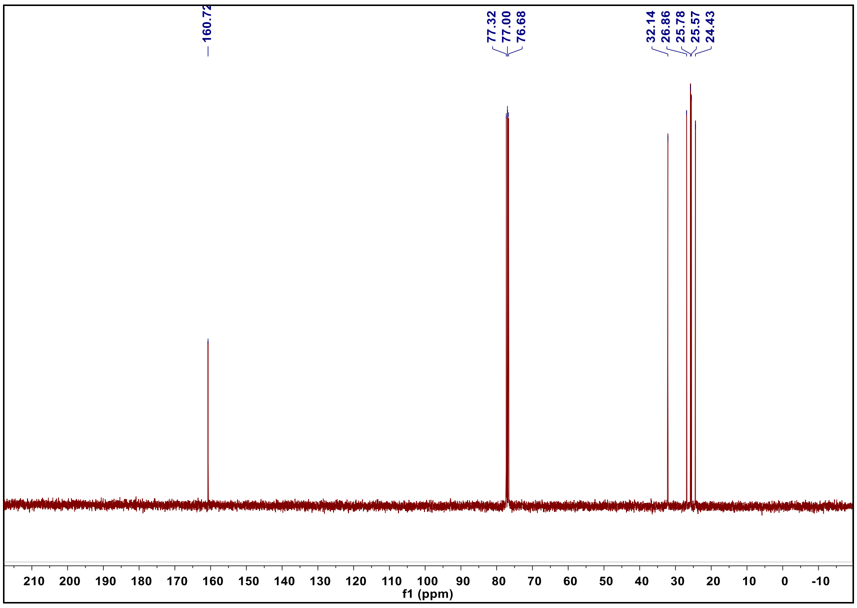

Supplementary Figure 7. ^{13}C NMR spectra of the cyclohexanone oxime product.

Supplementary Note 3. ^{13}C NMR (101 MHz, Chloroform-*d*) δ [ppm] 160.72, 32.14, 26.86, 25.78, 25.57, 24.43. GC/MS 113.0.

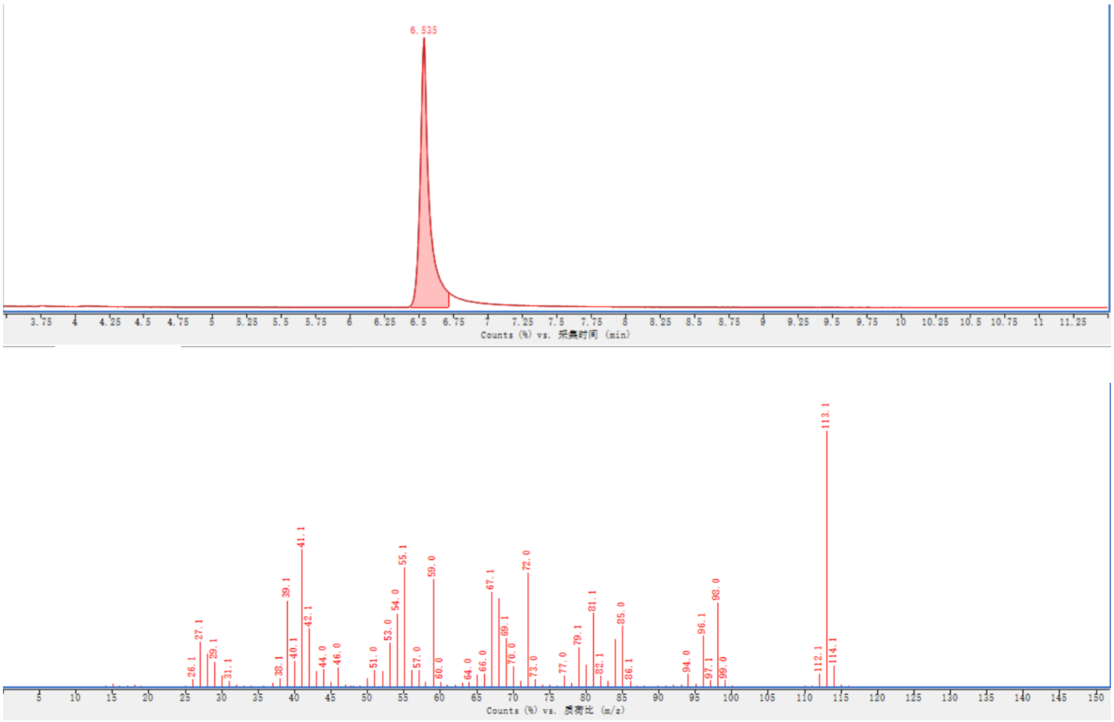

Supplementary Figure 8. GC–MS spectra of the reaction product obtained under standard conditions.

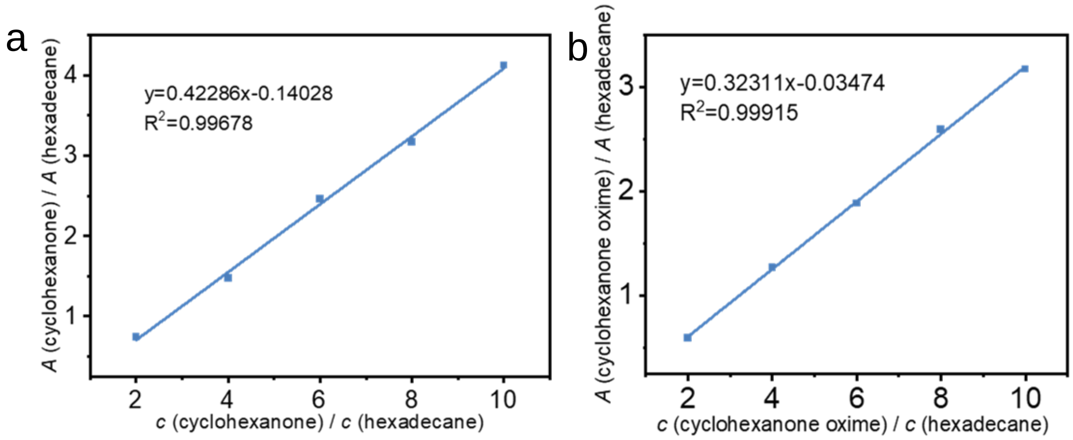

Supplementary Figure 9. The calibration curve for **a** cyclohexanone and **b** cyclohexanone oxime in the range of 0.00 mM~10 mM.

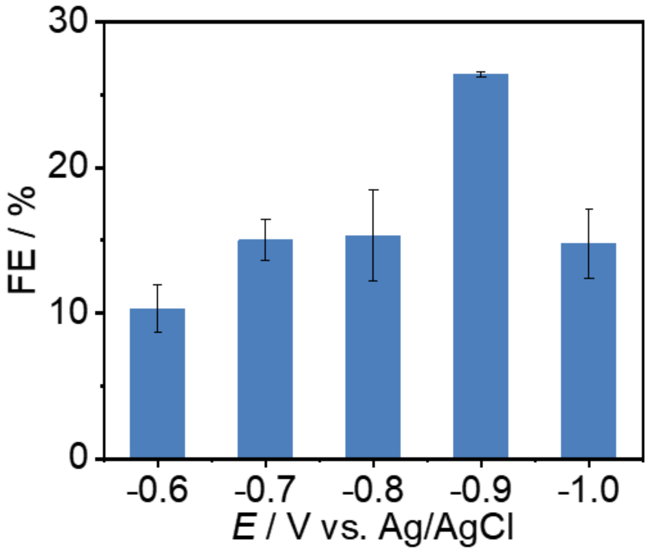

Supplementary Figure 10. Potential-dependent cyclohexanone oxime FE over a Cu-S cathode.

Supplementary Note 4. All the electrolysis tests at different potentials were conducted for 4000 s, and the amount of passed charge at potentials of -0.6, -0.7, -0.8, -0.9 and -1.0 V vs Ag/AgCl was 108, 174, 250, 275, and 496 C, respectively.

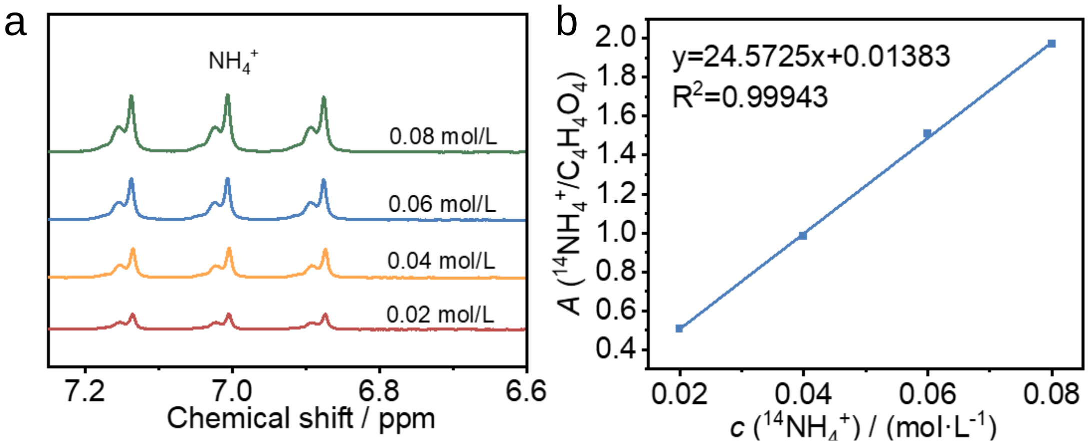

Supplementary Figure 11. Quantification of NH_4^+. a The ^1H NMR spectra of NH_4^+ standard samples with concentrations of 0.02 to 0.08 M and **b** the corresponding calibration curve.

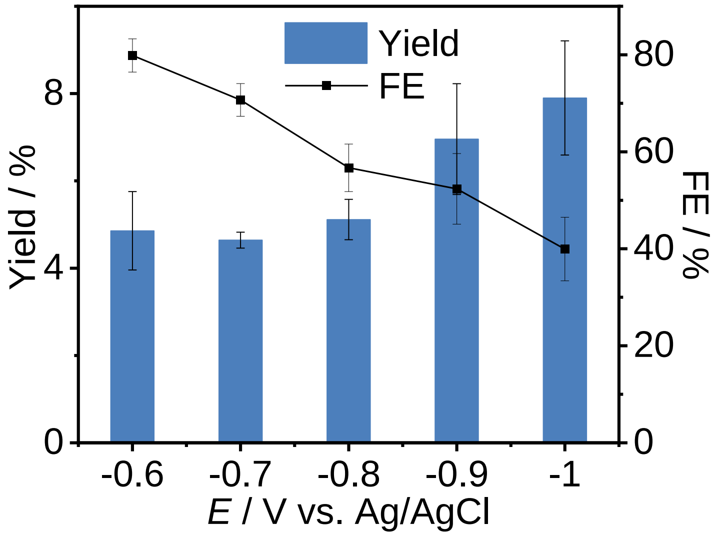

Supplementary Figure 12. Potential-dependent NH_4^+ yield and FE over a Cu-S cathode.

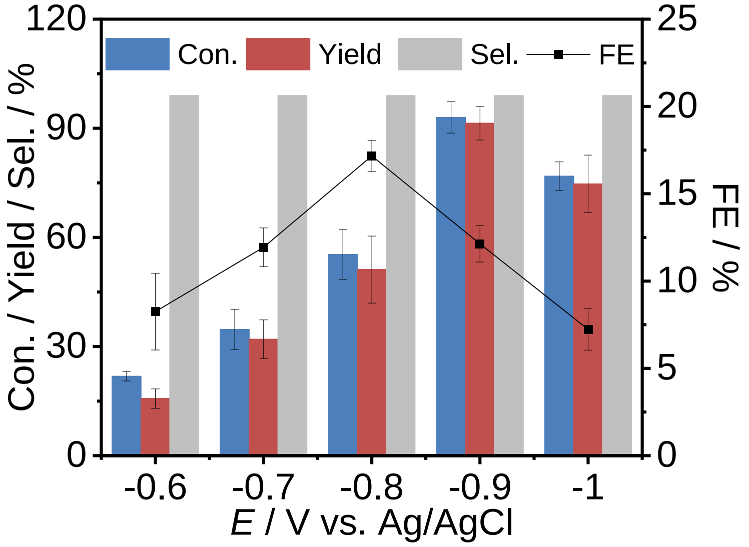

Supplementary Figure 13. Potential-dependent cyclohexanone conversion, cyclohexanone oxime yield, selectivity, and FE over a Cu cathode.

Supplementary Note 5. All the electrolysis tests at different potentials were conducted for 4000 s, and the amount of passed charge at potentials of -0.6, -0.7, -0.8, -0.9 and -1.0 V vs Ag/AgCl was 151, 213, 237, 600, and 822 C, respectively.

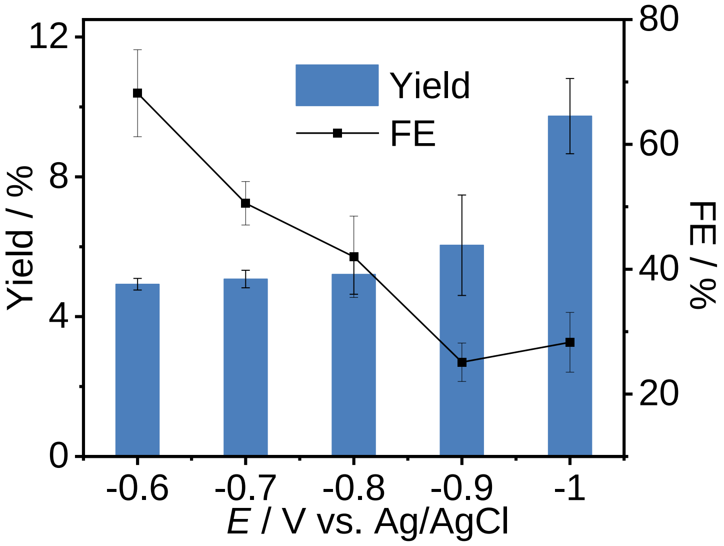

Supplementary Figure 14. Potential-dependent NH_4^+ yield and FE over a Cu cathode.

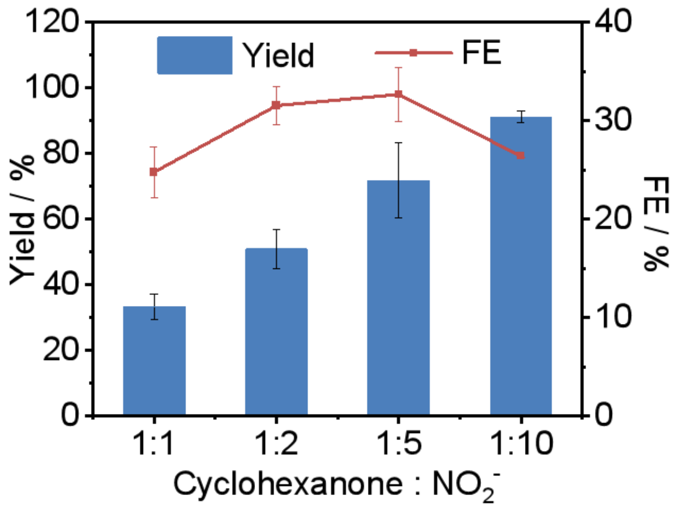

Supplementary Figure 15. Cyclohexanone oxime yield and FE under the conditions of different molar ratios of cyclohexanone and NO_2^-.

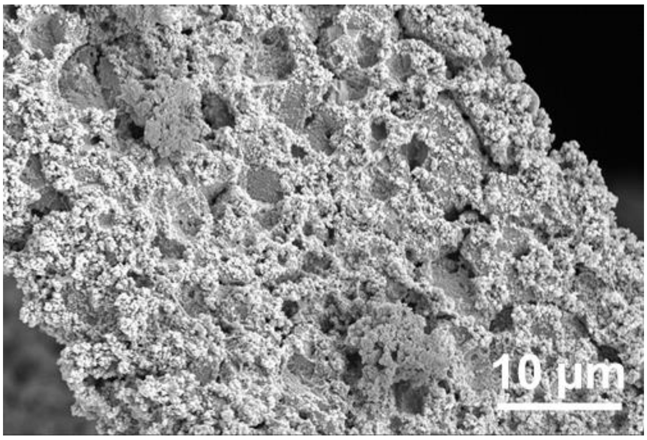

Supplementary Figure 16. SEM image of Cu-S catalyst after stability test.

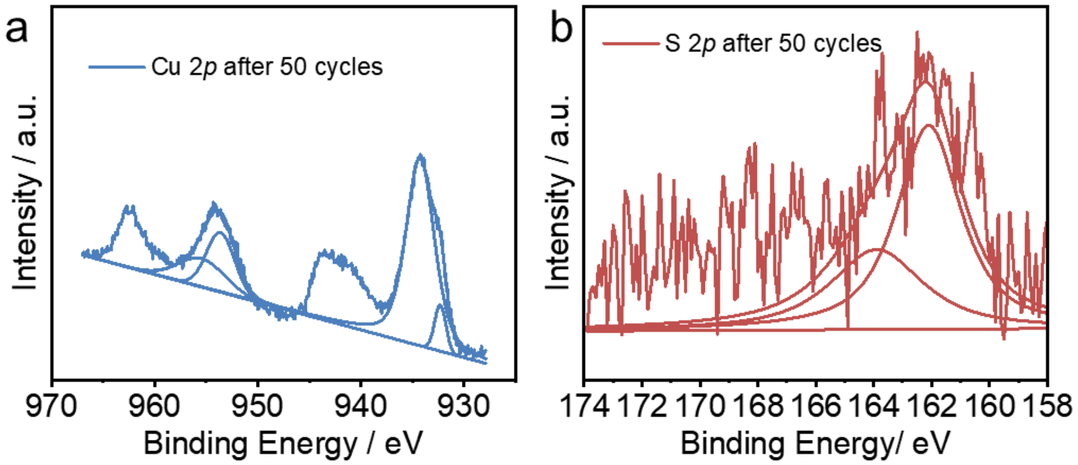

Supplementary Figure 17. Cu 2*p* and S 2*p* spectra of the Cu-S catalyst after the stability test.

Supplementary Note 6. The specific sample for the TEA calculation of cyclohexanone oxime electrosynthesis under optimistic case assumptions:

Taking the 50000 kg daily capacity of cyclohexanone oxime under a current density of 0.08 A cm^{-2} with 26% FE as an example. The corresponding calculation formulas are provided in the Excel file as Supplementary material.

The specific assumptions made for the TEA are listed as follows:

1. The operating voltage was calculated by the following equation according to the literature[2]:

$$Voltage = E_{\mathrm{ox}}^{0} - E_{\mathrm{re}} + E_1 + E_2 + E_3$$

where E^{0}_{ox} is the water oxide potential (1.23 V), E_{re} is the cathodic potential (–0.36 V), E_1 is the overpotential for water oxidation (0.6 V), and E_2 is the overpotential accounting for the impact of the membrane (1.0 V), and E_3 is the voltage occupied by the solution resistance. The voltage used in the TEA is 3.43 V.

2. According to the previous literature[3], the electrolyzer cost per area was calculated as:

$$\begin{aligned}\text{Electrolyzer cost per area}\\ &= 275.55\ \$\ \mathrm{kW}^{-1} \times \frac{0.08\ \mathrm{A}}{\mathrm{cm}^2} \times 3.43\ \mathrm{V} \times \frac{10^4\mathrm{cm}^2}{\mathrm{m}^2} \times \frac{\mathrm{kW}}{1000\mathrm{W}}\\ &= 756.1\ \$\ \mathrm{m}^2\end{aligned}$$

3. The anticipated service life of the electrolyzer was 10 years, and the working time per year was 350 days.
4. The cost of Balance of Plant was set as 58% of the cost of the electrolyzer[3-4].
5. The cost of separation equipment is assumed to be 20% of the electrolyzer cost.
6. The maintenance cost was set as 2.5% of the electrolyzer cost[4-5].
7. The separation cost is assumed to be 30% of the electricity cost[5].
8. For material costs, according to the experimental results, we rationally assumed that cyclohexanone could be fully converted into the target products. Thus, 0.876 tons of cyclohexanone and 6.097 tons of $NaNO_2$ were acquired to produce one ton of cyclohexanone oxime.
9. The electricity price was considered to be 0.1 $\$\ kWh^{-1}$(Ref.[5]).

Thus, the parameters needed in the cost calculation are determined as follows:

The required total current is calculated as:

$$\text{Total Current} = \frac{50000\ \text{kg}}{\text{day}} \times \frac{\text{day}}{86400\ \text{s}} \times \frac{1000\ \text{g}}{\text{kg}} \times \frac{\text{mol}}{28\ \text{g}} \times 4\text{e}^{-} \times \frac{96485\ \text{C}}{\text{mol}} \times \frac{1}{0.26}$$
$$= 7591322.53\ \ \text{A}$$

The needed electrolyzer area was determined as follows:

$$\text{Electrolyzer Area} = 7591322.53\ \ \text{A} \times \frac{1}{0.08\ \text{A cm}^{-2}} \times \frac{\text{m}^2}{10^4\ \text{cm}^2} = 9489.15\ \ \text{m}^2$$

The input power was calculated as:

$$\text{Power} = 3.43\ \text{V} \times 7591322.53\ \text{A} \times \frac{\text{W}}{10^6\ \text{MW}} = 26.04\ \text{MW}$$

The total cost of cyclohexanone oxime electrosynthesis consisted of capital cost, operating cost, and material cost.

Capital Cost:

1. Electrolyzer:

Electrolyzer Cost per ton Cyclohexanone oxime

$$= 9489.15\ \text{m}^2 \times 756.1\ \$\ \text{m}^{-2} \times \frac{1}{10\ \text{year}} \times \frac{\text{year}}{350\ \text{day}} \times \frac{\text{day}}{50\ \text{ton}}$$
$$= 41.00\ \$\ \text{ton}_{\text{oxime}}{}^{-1}$$

2. Balance of Plant:

Balance of Plant Cost per ton Cyclohexanone oxime

$$= 41.00\ \$\ \text{ton}_{\text{C}_2\text{H}_4}{}^{-1} \times 0.39/0.67 = 23.86\ \$\ \text{ton}_{\text{oxime}}{}^{-1}$$

3. Separation Equipment:

Separation Equipment Cost per ton Cyclohexanone oxime

$$= 41.00\ \$\ \text{ton}_{\text{oxime}}{}^{-1}\ \times 0.2 = 8.20\ \$\ \text{ton}_{\text{oxime}}{}^{-1}$$

Operating Cost:

1. Electricity:

$$26.04\ \text{MW} \times \frac{1000\ \text{kW}}{\text{MW}} \times 24\frac{\text{h}}{\text{day}} \times 0.1\frac{\$}{\text{kWh}} \times \frac{1\ \text{day}}{50\ \text{ton}} = 1249.84\ \$\ \text{ton}_{\text{oxime}}{}^{-1}$$

2. Maintenance:

$$41.00\ \$\ \text{ton}_{\text{oxime}}{}^{-1} \times 0.025 = 1.02\ \$\ \text{ton}_{\text{oxime}}{}^{-1}$$

3. Separation:

$$1249.84\ \$\ ton_{oxime}{}^{-1} \times 0.3 = 374.95\ \$\ \text{ton}_{\text{oxime}}{}^{-1}$$

Materials Cost:

The market price of cyclohexanone is 1161 \$ ton^{-1} according to the recently published literature[14]. Thus, the cost of cyclohexanone was calculated as follows:

$$1161\ \$\ \mathrm{ton_{cyclohexanone}}^{-1} \times 0.876 = 1017.17\ \$\ \mathrm{ton_{oxime}}^{-1}$$

Based on the data on the website named Zauba (Ref. 13) aimed at providing guidance for import and export trade, the price of $NaNO_2$ is 592.49 \$ ton^{-1}. The cost of $NaNO_2$ was calculated as follows:

$$592.49\ \$\ \mathrm{ton_{NaNO2}}^{-1} \times 6.097 = 3612.38\ \$\ \mathrm{ton_{oxime}}^{-1}$$

The total cost:

$$\text{Total cost} = (41.00 + 23.86 + 8.2 + 1249.84 + 1.02 + 374.95 + 1017.17 + 3612.38) = 6328.04\ \$\ \mathrm{ton_{oxime}}^{-1}$$

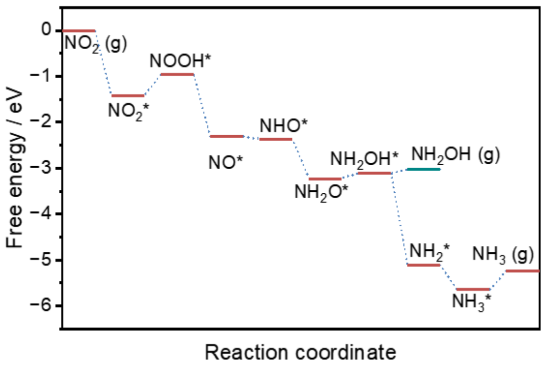

Supplementary Figure 18. Free energy diagram for cyclohexanone oxime generation over a Cu-S cathode.

Supplementary Note 7. All calculations are performed using the Vienna ab initio simulation package (VASP) based on density functional theory (DFT)[6]. Projector augmented wave (PAW) pseudopotentials are employed[7]. The exchange-correlation contributions to the total energy are estimated by the generalized gradient approximation (GGA) with the Perdew–Burke–Ernzerhof (PBE) form. The Hubbard U approach (DFT+U) is adopted to better describe the on-site Coulomb correlation of the localized 3d electrons for Cu with $U - J = 3.87$ eV[8-10]. An empirical dispersion-corrected DFT method (DFT-D_3) is carried out to reasonably describe the weak long-distance van der Waals effects[11]. The solvent effect (G_{solv}) is considered for thermodynamic corrections by using VASPsol[12]. The kinetic energy cut-off for plane-wave expansion is set to 500 eV. An energy convergence threshold of 10^{-4} eV is set in the self-consistent field iteration. Geometry optimization within the conjugate gradient method is performed with forces on each atom < 0.05 eV Å^{-1}. A p (2 × 2) slab model with three atomic layers is adopted to simulate the Cu (111) surface. One of the surface Cu atoms is replaced by an S atom to represent the S-doped Cu surface. A vacuum layer of 15 Å is inserted along the c direction to eliminate the periodic image interactions. The bottom atomic layer is fixed, while other layers and the adsorbates are fully relaxed during structural optimizations. The Brillouin zone is sampled by a k-point mesh of 4 × 4 × 1. The reaction free energy change can be obtained with equation (1):

$$\Delta G = \Delta E + \Delta E_{ZPE} - T\Delta S$$

where ΔE is the total energy difference between the products and the reactants of each reaction step, and ΔE_{ZPE} and ΔS are the differences in zero-point energy and entropy, respectively. The zero-point energies of free molecules and adsorbates are obtained from vibrational frequency calculations. The free energy change of each step that involves electrochemical proton-electron transfer is described by the computational hydrogen electrode (CHE) model proposed by Nørskov et al[13]. In this technique, zero voltage is defined based on the reversible hydrogen electrode, in which the reaction is defined to be in equilibrium at zero voltage, at all pH values, at all temperatures, and with H_2 at 101,325 Pa pressure. Therefore, in the CHE model, the free energy of a proton-electron pair is equal to half of the free energy of gaseous hydrogen at a potential of 0 V.

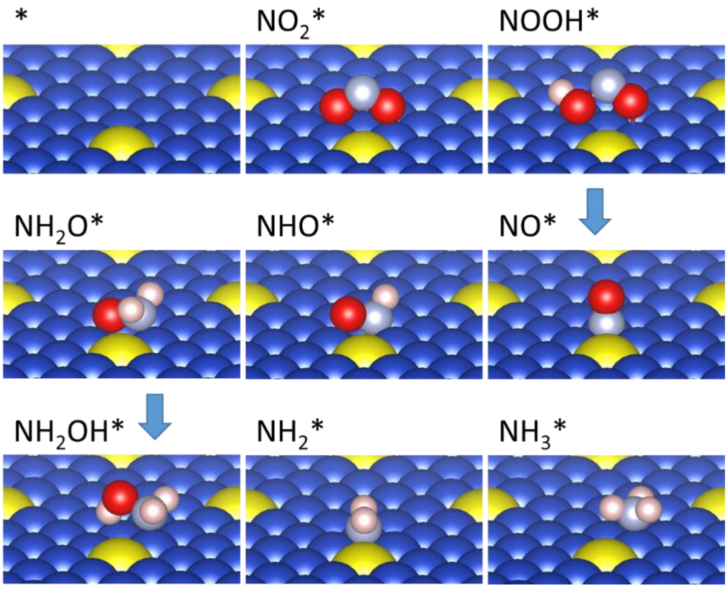

Supplementary Figure 19. The optimized adsorption geometries of NO_2*, NOOH*, NO*, NHO*, NH_2O*, NH_2OH*, NH_2* and NH_3* on the Cu-S surface.

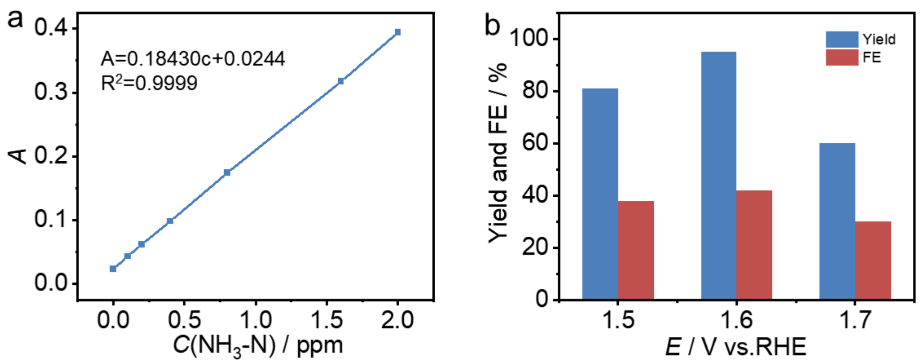

Supplementary Figure 20. (a) The calibration curves of NH_3-N based on the absorbance of different ion concentrations[1] and (b) the FEs and yields of NO_2^- for NH_4^+ electrooxidation at different potentials over a $Cu(OH)_2$ electrode.

Supplementary Note 8. The electrooxidation of NH_4^+ to NO_2^- is conducted in a two-compartment three-electrode system. 0.1 M KOH solution containing 10 mM NH_4Cl was used as the electrolyte. $Cu(OH)_2$ supported on Cu foam, denoted $Cu(OH)_2$/Cu NF, was used as the working electrode. The electrolytic reaction proceeded at potentials of 1.5 to 1.7 V vs. RHE. The produced NH_4^+ was quantified by the UV–vis absorption spectrum after passing a charge of 260 C. Impressively, 42% FE and 95% yield of NH_4^+ were obtained at the optimal potential of 1.6 V vs. RHE, rationalizing the recycling idea of the N source.

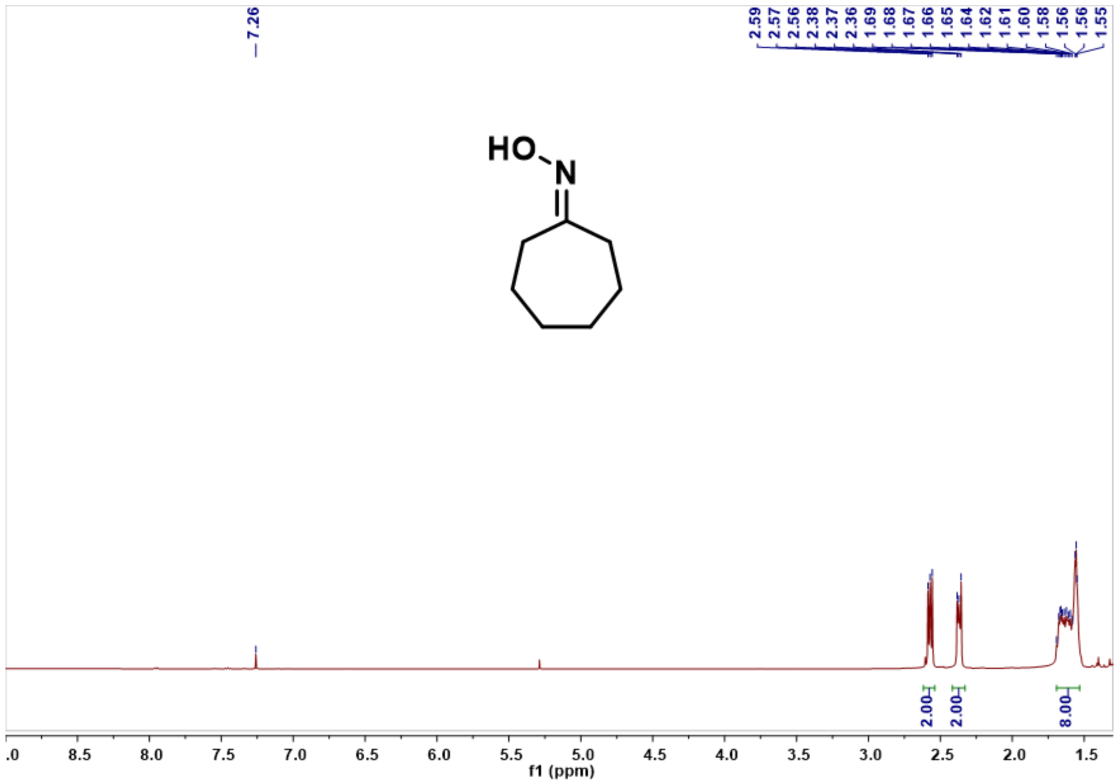

Supplementary Figure 21. ^{1}H NMR spectrum of cycloheptanone oxime.

Supplementary Note 9. ^{1}H NMR (400 MHz, Chloroform-*d*) δ [ppm] 2.57 (t, *J* = 6.0 Hz, 2H), 2.37 (t, *J* = 6.0 Hz, 2H), 1.70-1.55 (m, 8H). GC/MS 127.1.

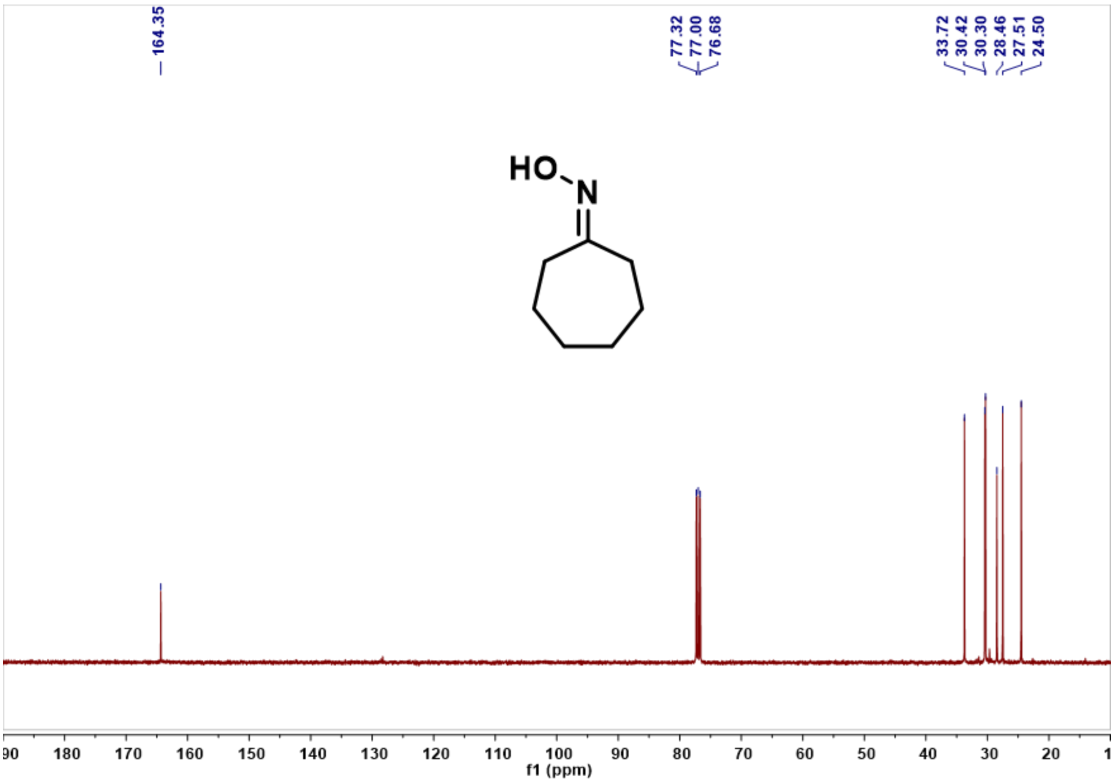

Supplementary Figure 22. ^{13}C NMR spectrum of cycloheptanone oxime.

Supplementary Note 10. ^{13}C NMR (101 MHz, Chloroform-*d*) δ [ppm] 164.35, 33.72, 30.42, 30.30, 28.46, 27.51, 24.50. GC/MS 127.1.

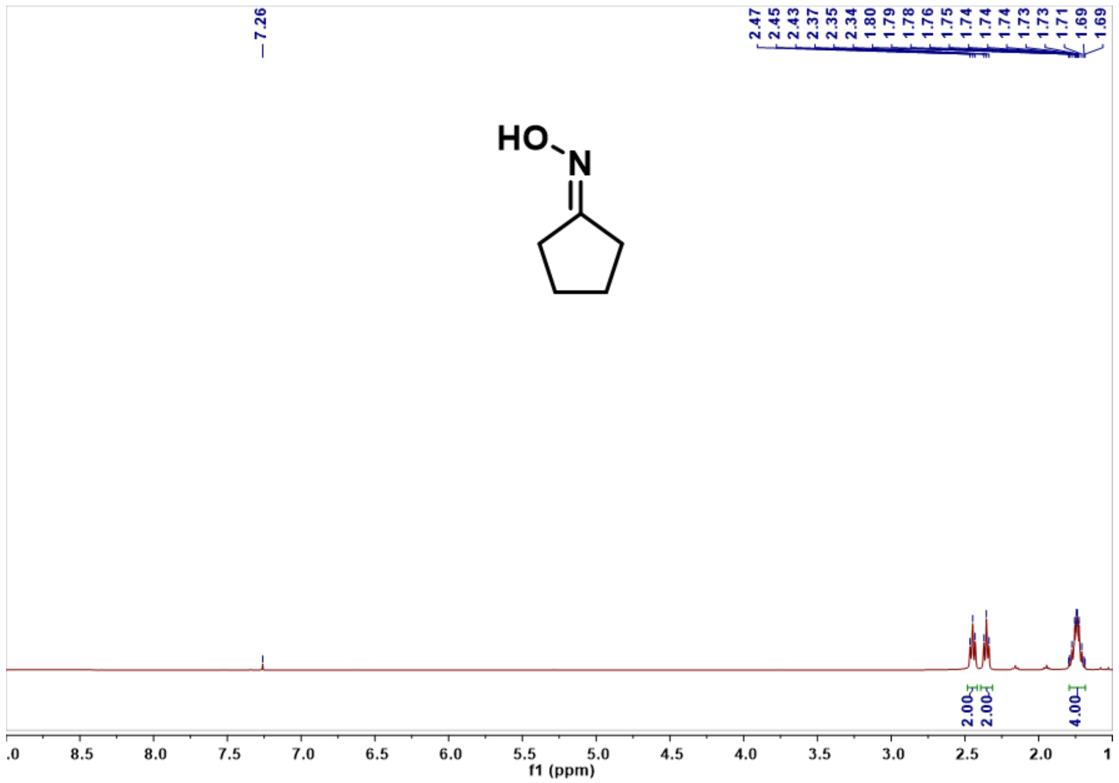

Supplementary Figure 23. ^{1}H NMR spectrum of cyclopentanone oxime.

Supplementary Note 11. ^{1}H NMR (400 MHz, Chloroform-*d*) δ [ppm] 2.45 (t, *J* = 6.4 Hz, 2H), 2.36 (t, *J* = 6.4 Hz, 2H), 1.80-1.68 (m, 4H). GC/MS 99.0

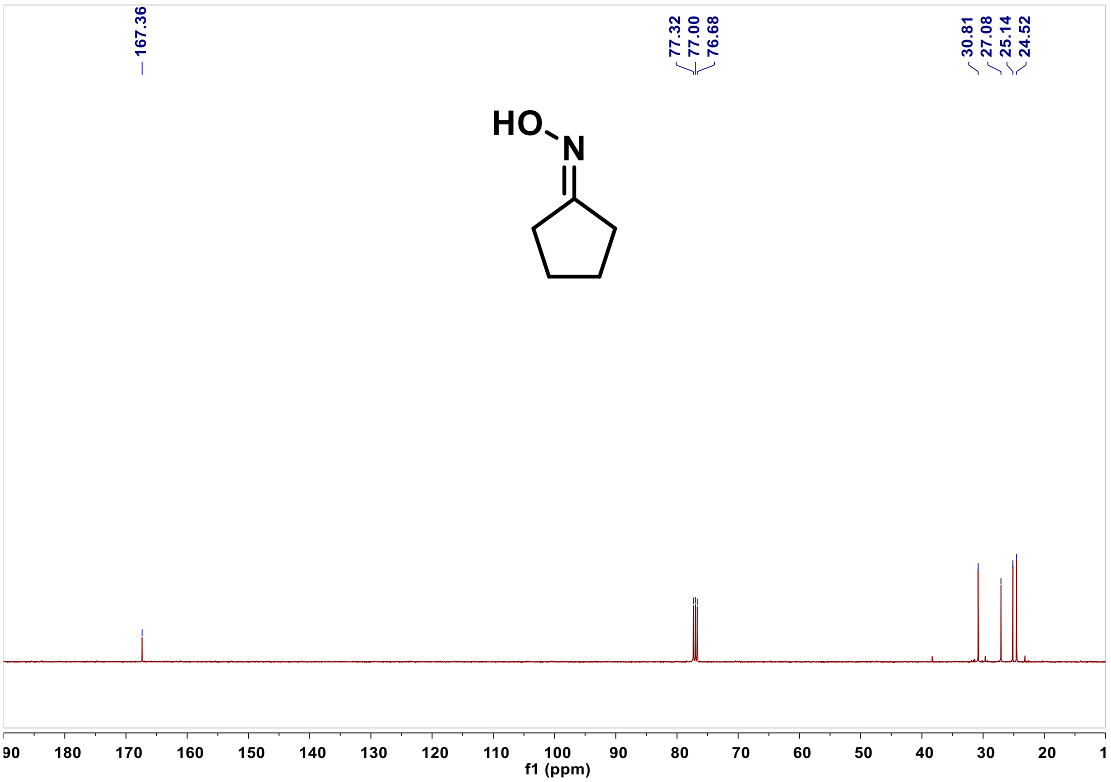

Supplementary Figure 24. ^{13}C NMR spectrum of cyclopentanone oxime.

Supplementary Note 12. ^{13}C NMR (101 MHz, Chloroform-*d*) δ [ppm] 167.36, 30.81, 27.08, 25.14, 24.52. GC/MS 99.0

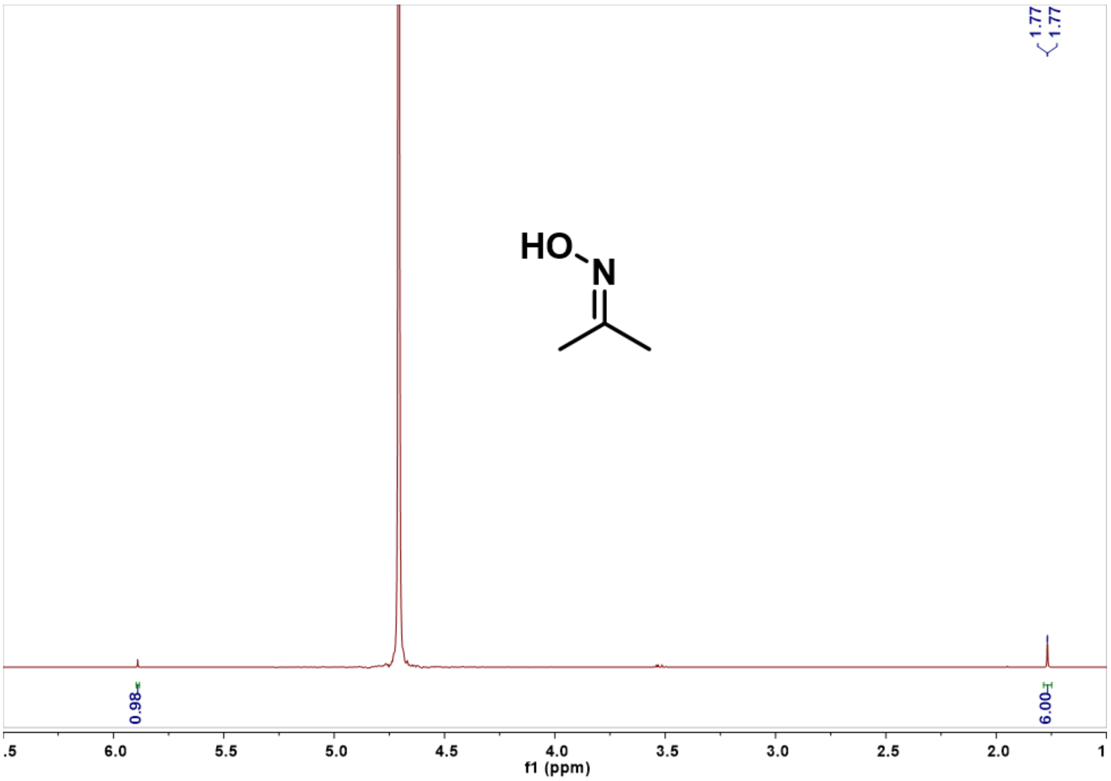

Supplementary Figure 25. ^{1}H NMR spectrum of acetone oxime.

Supplementary Note 13. ^{1}H NMR (400 MHz, Chloroform-*d*) δ [ppm] 1.77 (d, *J* = 0.8 Hz, 6H). The peak at 5.89 ppm belongs to the maleic acid standard. GC/MS 73.0.

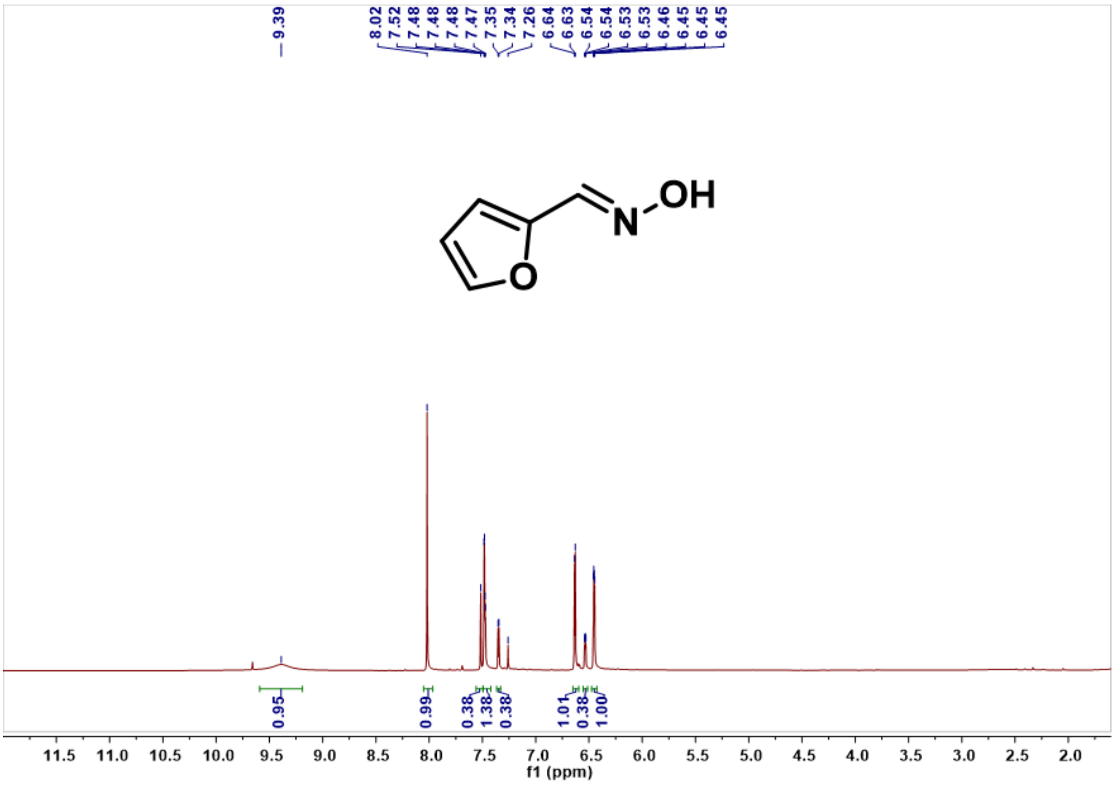

Supplementary Figure 26. ^{1}H NMR spectrum of furfural oxime.

Supplementary Note 14. ^{1}H NMR (400 MHz, Chloroform-*d*) δ [ppm] 9.39 (s, 0.95H), 8.02 (s, 1H), 7.52 (s, 0.38H), 7.49-7.47 (m, 1.38H), 7.35 (d, J = 3.6 Hz, 0.38H), 6.63 (d, J = 3.2 Hz, 1H), 6.54 (m, 0.38H), 6.45 (m, 1H). GC/MS 111.0.

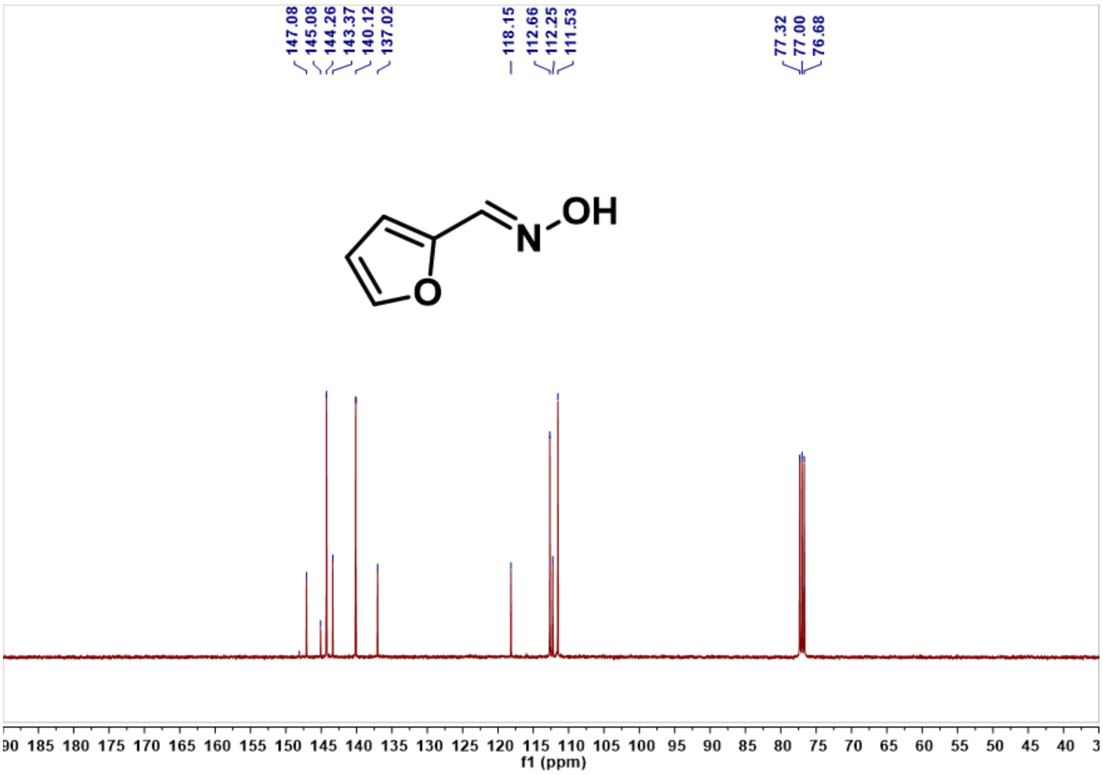

Supplementary Figure 27. ^{13}C NMR of furfural oxime.

Supplementary Note 15. ^{13}C NMR (101 MHz, Chloroform-*d*) δ [ppm] 147.08, 145.08, 144.26, 143.37, 140.12, 137.02, 118.15, 112.66, 112.25, 111.53. GC/MS 111.0.

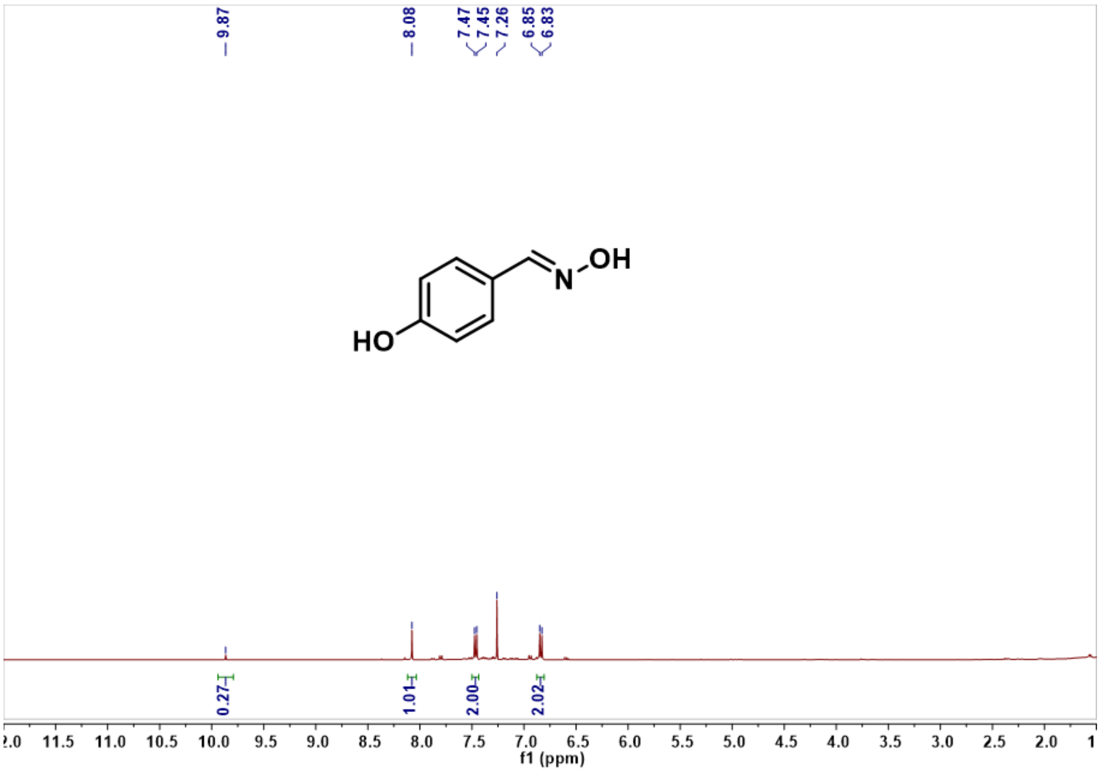

Supplementary Figure 28. ^{1}H NMR spectrum of 4-hydroxybenzaldehyde oxime.

Supplementary Note 16. ^{1}H NMR (400 MHz, Chloroform-*d*) δ [ppm] 9.87 (s, 0.27H), 8.08 (s,1H), 7.46 (d, *J* = 8.8 Hz, 2H), 6.84 (d, *J* = 8.8 Hz, 2H). GC/MS 137.0.

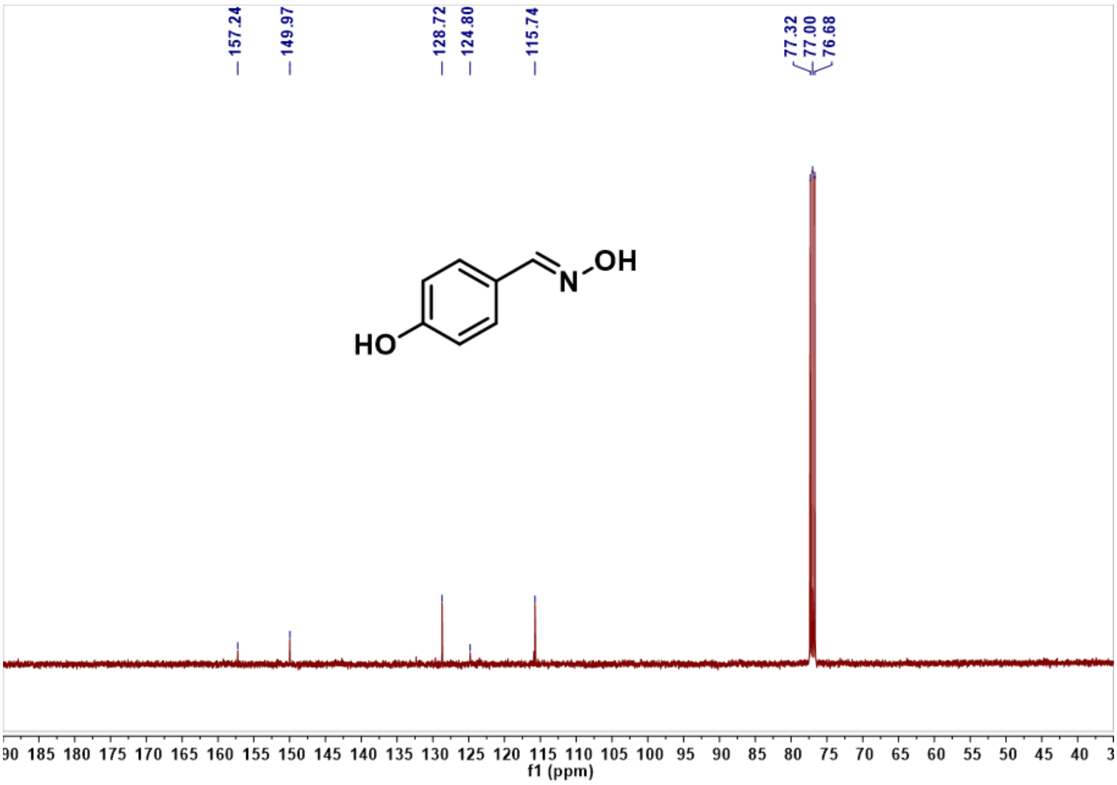

Supplementary Figure 29. ^{13}C NMR spectrum of 4-hydroxybenzaldehyde oxime.

Supplementary Note 17. ^{13}C NMR (101 MHz, Chloroform-*d*) δ [ppm] 157.24, 149.97, 128.72, 124.80, 115.74. GC/MS 137.0.

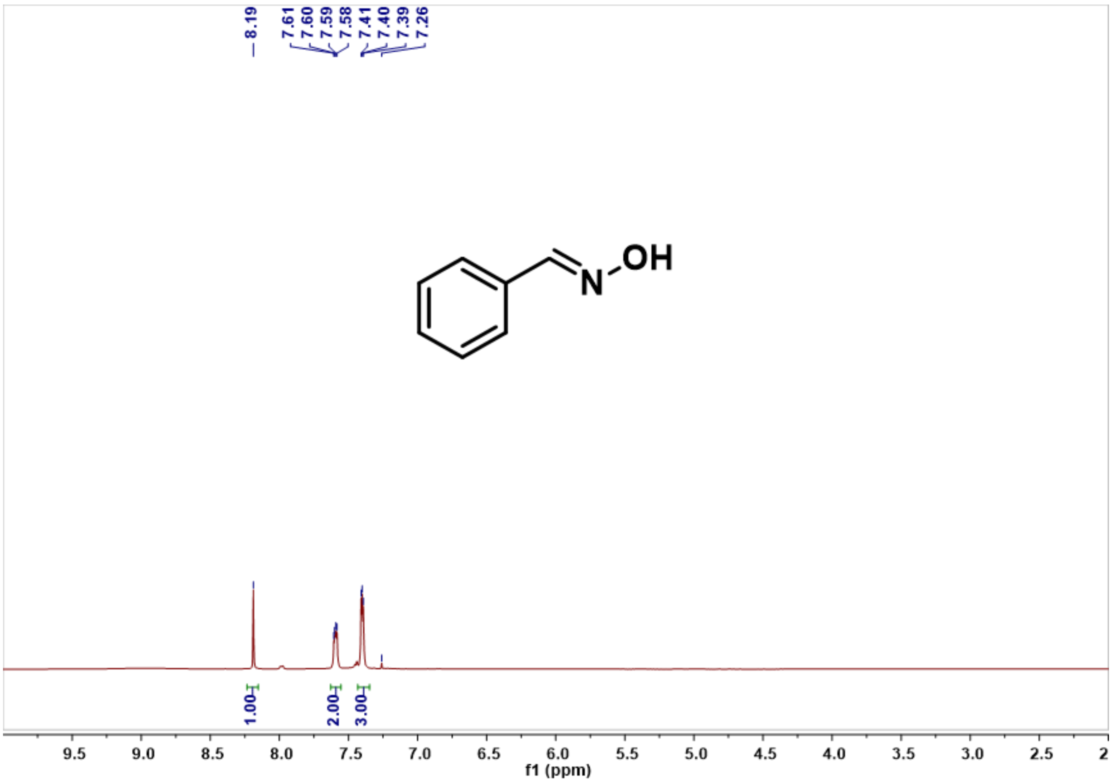

Supplementary Figure 30. ^{1}H NMR spectrum of benzaldehyde oxime.

Supplementary Note 18. ^{1}H NMR (400 MHz, Chloroform-*d*) δ [ppm] 8.19 (s, 1H), 7.60 (dd, *J* = 3.2 Hz, 2H), 7.40 (t, *J* = 3.2 Hz, 3H). GC/MS 121.0.

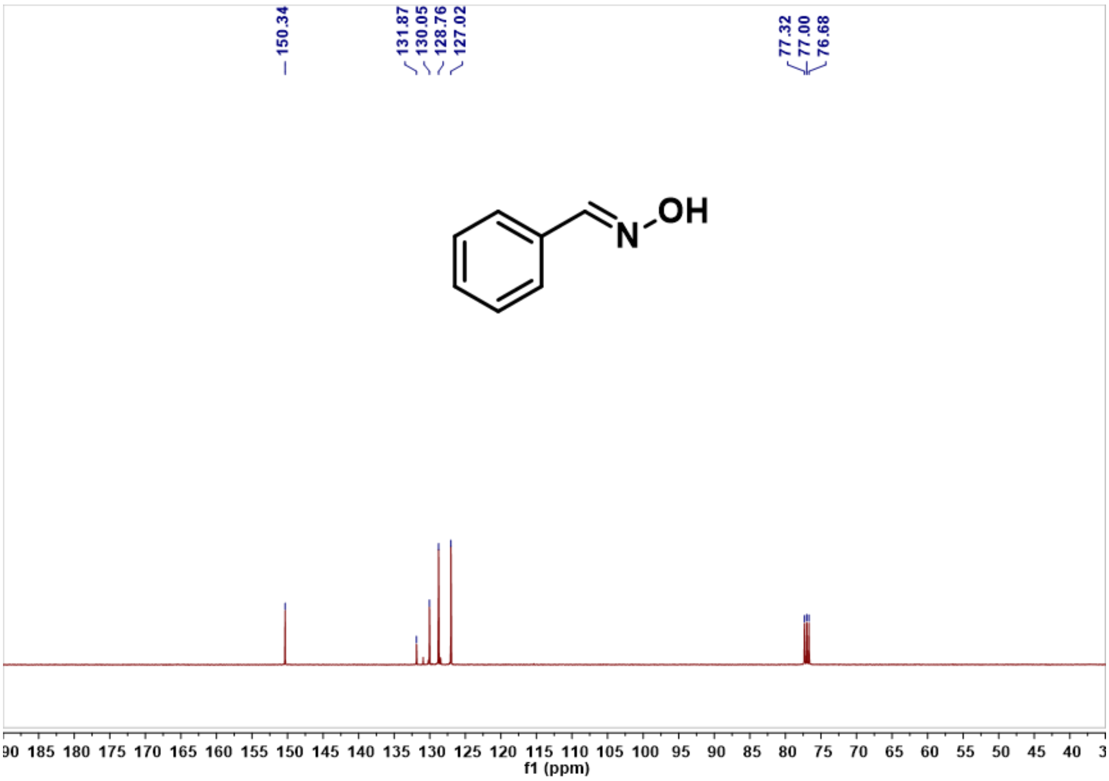

Supplementary Figure 31. ^{13}C NMR spectrum of benzaldehyde oxime.

Supplementary Note 19. ^{13}C NMR (101 MHz, Chloroform-*d*) δ [ppm] 150.34, 131.87, 130.05, 128.76, 127.02. GC/MS 121.0.

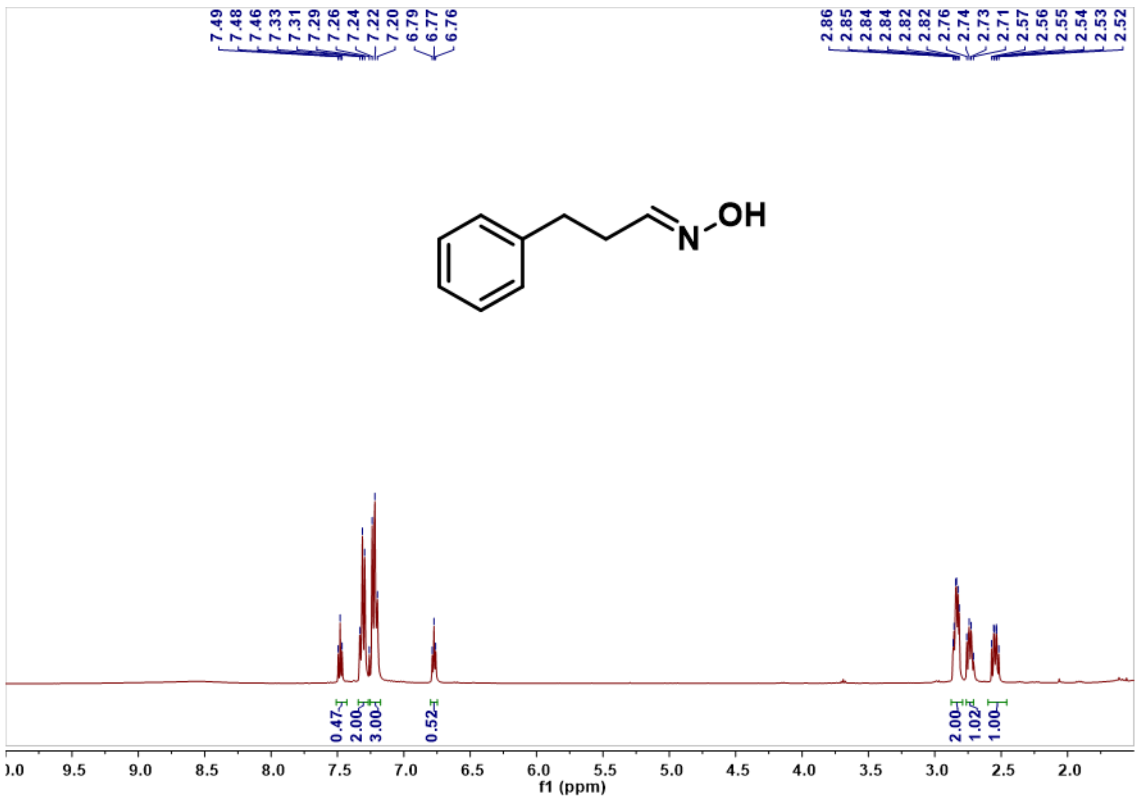

Supplementary Figure 32. ^{1}H NMR spectrum of phenylpropyl aldehyde oxime.

Supplementary Note 20. ^{1}H NMR (400 MHz, Chloroform-*d*) δ [ppm] 7.48 (t, *J* = 5.6 Hz, 0.47H), 7.34-7.29 (m, 2H), 7.27-7.19 (m, 3H), 6.77 (t, *J* = 5.2 Hz, 0.52H), 2.87-2.81 (m, 2H), 2.76-2.70 (m, 1H), 2.58-2.51 (m, 1H). GC/MS 149.0.

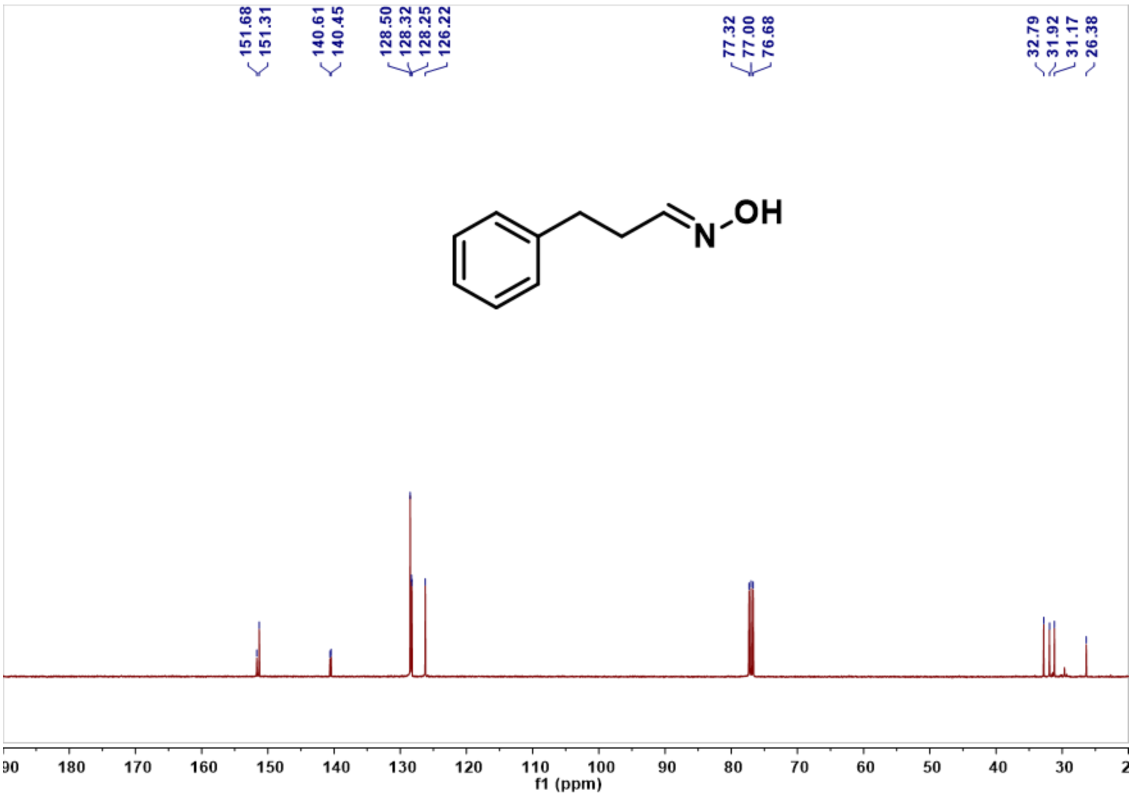

Supplementary Figure 33. ^{13}C NMR spectrum of phenylpropyl aldehyde oxime.

Supplementary Note 21. ^{13}C NMR (101 MHz, Chloroform-*d*) δ [ppm] 151.68, 151.31, 140.61, 140.45, 128.50, 128.32, 128.25, 126.22, 32.79, 31.92, 31.17, 26.38. GC/MS 149.0.

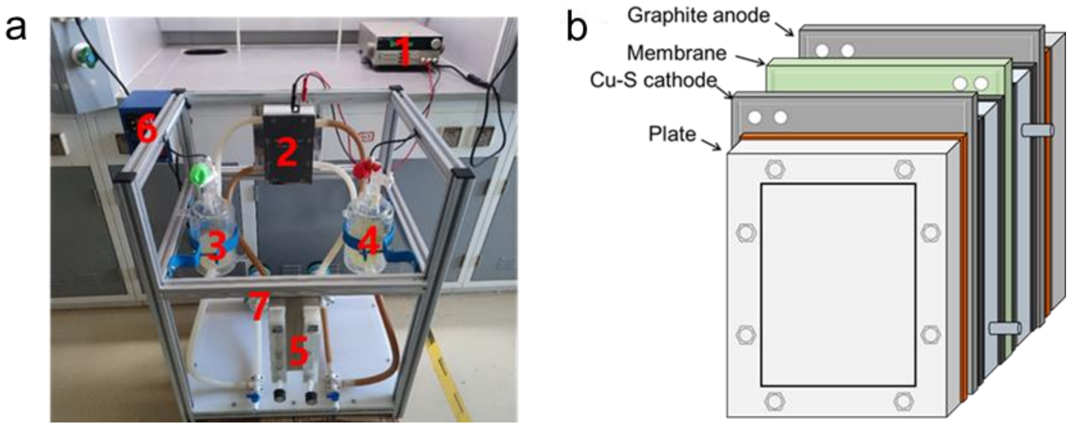

Supplementary Figure 34. a Photograph of the flow reactor setup and b schematic illustration of the electrolyzer.

Supplementary Note 22. The assembled flow cell was connected by a fluororubber tube to a magnetic circulating pump and a 1.0 L jacketed glass reservoir. A flow meter (measuring range from 16 L/h to 160 L/h) was placed between the assembled cell and pump. The Teflon barbed fittings on the side of PTFE frames were connected via a fluororubber tube to the fitting on the adjacent PTFE frame to allow the solution to flow between each cell. One of the two end fittings connects to the pump, as the inlet of flow transferring reaction solution from reservoir to the cell; another fitting was connected directly to the reservoir, as the outlet of flow moving solution from the cell back to the reservoir and thus forming a closed flow loop. The cathode plate is connected through a wire to the negative terminal of a direct current power supply, while the corresponding anode plates are connected to the positive terminal. The parts of the setup are marked in the photograph: 1-direct current power; 2-electrolyzer; 3-anode reservoir; 4-cathode reservoir; 5-flow meter; 6-thermo detector; 7-magnetic circulating pump.

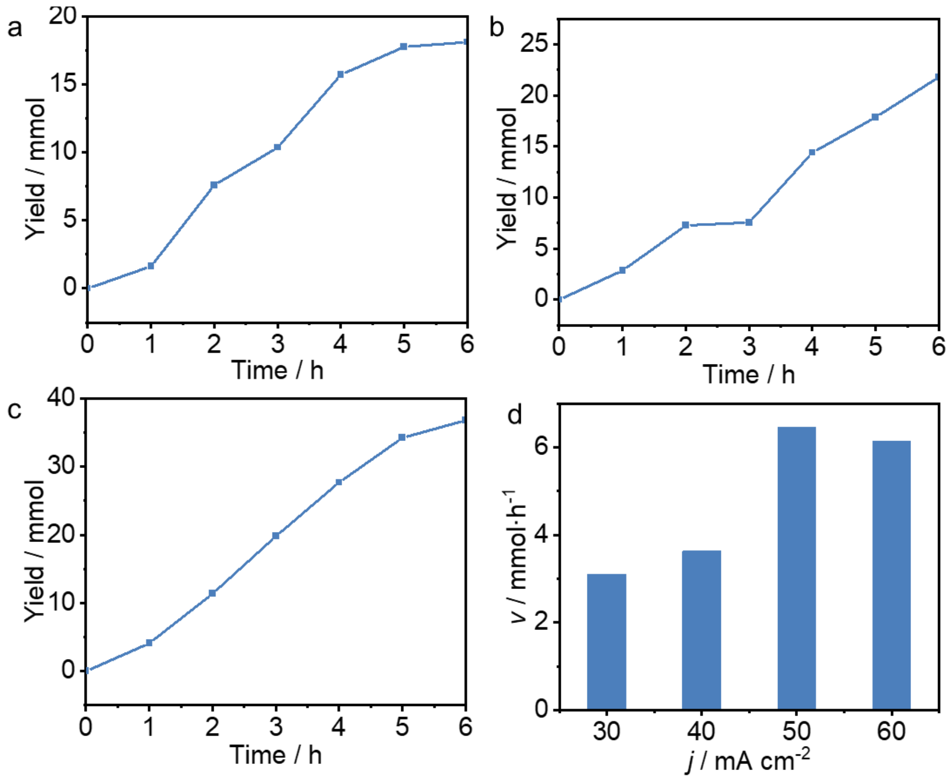

Supplementary Figure 35. Time-dependent cyclohexanone oxime yield at current densities of (A) 30, (B) 40, and (C) 60 mA cm^{-2} and (D) cyclohexanone oxime production rate at different current densities.

Supplementary References

1. Lewis, R. J. et al. Highly efficient catalytic production of oximes from ketones using in situ-generated H_2O_2. *Science* **376**, 615–620 (2022).
2. Huang, Y., Wang, Y., Wu, Y., Yu, Y. & Zhang, B. Electrocatalytic construction of the C-N bond from the derivates of CO_2 and N_2. *Sci China Chem* **65**, 204–206 (2022).
3. Jouny, M., Luc, W. & Jiao, F. General techno-economic analysis of CO_2 electrolysis systems. *Ind Eng. Chem. Res.* **57**, 2165–2177 (2018).
4. Lum, Y. et al. Tuning OH binding energy enables selective electrochemical oxidation of ethylene to ethylene glycol. *Nat. Catal.* **3**, 14–22 (2020)
5. ZAUBA, Detailed Export Data of sodium nitrite. https://www.zauba.com/export-sodium-nitrite-hs-code.html.
6. Kresse, G. & Furthmüller, J. Efficient iterative schemes for ab initio total-energy calculations using a plane-wave basis set. *Phys. Rev. B* **54**, 11169–11186 (1996).
7. Kresse, G. & Furthmüller, J. Efficiency of ab-initio total energy calculations for metals and semiconductors using a plane-wave basis set. *Comp. Mater. Sci.* **6**, 15–50 (1996).
8. G. Kresse, J. Furthmüller, Efficient iterative schemes for ab initio total-energy calculations using a plane-wave basis set. *Phys. Rev. B* **54**, 11169–11186 (1996).
9. Dudarev, S. L., Botton, G. A., Savrasov, S. Y., Humphreys, C. J. & Sutton,A. P. Electron-energy-loss spectra and the structural stability of nickel oxide: an LSDA+U study. *Phys. Rev. B* **57**, 1505–1509 (1998).
10. Xu, H., Cheng, D., Cao, D. & Zeng, X. A universal principle for a rational design of single-atom electrocatalysts. *Nat. Catal.* **1**, 339–348 (2018)
11. Liu, X. C. et al. Cathode-introduced atomic H* for Fe (II)-complex regeneration to effective electro-fenton process at a natural pH. *Environ. Sci. Technol*. **53**, 6927–6936 (2019)
12. Mathew, K., Sundararaman, R., Letchworthweaver, K., Arias, T. A. & Hennig, R. G. Implicit solvation model for density-functional study of nanocrystal surfaces and reaction pathways. *J. Chem. Phys.* **140**, 084106 (2014).
13. Nørskov, J., Rossmeisl, J., Logadottir, A. & Lindqvist, L. Origin of the overpotential for oxygen reduction at a fuel-cell cathode. *J. Phys. Chem. B* **108**, 17886–17892 (2004).
